# Analytical study of one dimensional time fractional Schrödinger problems arising in quantum mechanics

**DOI:** 10.1038/s41598-024-63286-3

**Published:** 2024-05-31

**Authors:** Muhammad Nadeem, Yahya Alsayaad

**Affiliations:** 1 School of Mathematics and Statistics, Qujing Normal University, Qujing, 655011 China; 2 Department of Physics, Hodeidah University, Al-Hudaydah, Yemen

**Keywords:** Sumudu transform, Residual power series method, Time-fractional schrödinger equation, Series solution, Applied mathematics, Computational science, Software

## Abstract

This work presents the analytical study of one dimensional time-fractional nonlinear Schrödinger equation arising in quantum mechanics. In present research, we establish an idea of the Sumudu transform residual power series method (ST-RPSM) to generate the numerical solution of nonlinear Schrödinger models with the fractional derivatives. The proposed idea is the composition of Sumudu transform (ST) and the residual power series method (RPSM). The fractional derivatives are taken in Caputo sense. The proposed technique is unique since it requires no assumptions or variable constraints. The ST-RPSM obtains its results through a series of successive iterations, and the resulting form rapidly converges to the exact solution. The results obtained via ST-RPSM show that this scheme is authentic, effective, and simple for nonlinear fractional models. Some graphical structures are displayed at different levels of fractional orders using Mathematica Software.

## Introduction

Numerous phenomena in nature demonstrate intricate, non-linear patterns of behavior that can not be sufficiently represented by conventional integer-order versions. Fractional calculus offers a more precise conceptual structure for characterizing these systems and provides a better depiction of their dynamics. In recent decades, fractional calculus has increased interest in a variety of science and engineering problems. Fractional partial differential equations (FPDEs) make it easy to solve numerous types of physical phenomena, notably elasticity, bloodstream fluid, solid geometry, optic fibers, processing of signals, radiation, hydrodynamics, medical science, and the process of diffusion^1–5^. The majority of FPDEs can not be solved precisely. Numerous scientists have tried a variety of strategies to obtain the accurate solutions of FPDEs, such as the Collocation method^6^, Local fractional natural decomposition method^7^, Extrapolated Crank-Nicolson scheme^8^, Variational scheme^9^, Homotopy analysis approach^10^, Homotopy asymptotic strategy^11^, Homotopy perturbation approach^12^, Natural homotopy perturbation method^13^, Orthogonal spline collocation^14^, Differential transform scheme^15^, Finite difference strategy^16^, and Adomian decomposition technique^17^.

In the domain of quantum study, the time-fractional Schrödinger equation (SE) is an extension of traditional SE, that originates from fractional theories of quantum mechanics. The NLSE of fractional order $\alpha$ such that $0< \alpha < 1$ involves the time derivative but the conventional NLSE contains the first-order time derivative. Therefore, the fractional SE represents a partial differential equation (PDE) of fractional order $\alpha$ in coherent with recent usage. The scientific concept explaining the structure of induced findings for time-fractional NLSE is significant and impressive in the fields of quantum research, statistics, and advances in technology^18–20^. The prevalent and widely applicable time-fractional NLSE in a one-dimensional form is^21^

1 $$\begin{aligned} i D_\tau ^\alpha \mathfrak {S}(\aleph , \wp )+\sigma \mathfrak {S}_{\aleph \aleph }(\aleph , \wp )+\sigma |\mathfrak {S}(\aleph , \wp )|^2 \mathfrak {S}(\aleph , \wp )+\varsigma (\aleph )\mathfrak {S}(\aleph , \wp )=0,\quad \aleph \in \mathbb {R},\ \wp \ge 0,\ 0<\alpha \le 1, \end{aligned}$$

along subsequent condition

2 $$\begin{aligned} \mathfrak {S}(\aleph , 0)=\varrho (\aleph ), \end{aligned}$$

where $i^{2}=-1$, $\sigma \in \mathbb {R}$ is constant, $\mid .\mid$ is modulus, and $D_{t}^{\alpha }$ shows the derivative of order of $\alpha$ in time *t*. The $\mathfrak {S}$ is the waveform, $\varsigma (\aleph )$ is a function in analytical form, and $\varrho (\aleph )$ shows the function of displacement.

The analysis of the time-fractional NLSE is a significant dynamic topic within the field of fractional quantum physics. Most of the time, the analytical solution of NLSE is a challenging task and their obtained results are difficult to show in a closed-form expression, thus the solution of such a problem always requires a physical attraction. Numerous researchers have used multiple approaches to improve the computational outcomes of time fractional NLSE in practical applications. Sadighi and Ganji^22^ utilized a perturbation strategy and decomposition scheme to calculate the approximation of traditional SE. The authors in^23^ used RPSM to compute the solution of time-fractional NLSE and obtained the results in convergence series near the precise results. In^24^, the authors obtained the analytical results of NLSE in the Caputo sense by using Laplace transform homotopy analysis scheme. Liaqat and Akgül^25^ considered a natural-based perturbation scheme to derive the analytical results on this model. Khan et al.^26^ utilized the homotopy analysis approach to solve both the SE and the coupled SE. Okposo et al.^27^ introduced a *q*-homotopy analysis transform method to derive analytical findings for a system of nonlinear coupled SE that includes a time-fractional derivative in the Caputo sense.

The Sumudu transformation method, established in the early 1990s, is a significant transformation technique. It is an effective tool for resolving a wide variety of FPDEs in numerous scientific and engineering domains. The ST-RPSM provides better performance than conventional RPSM since the Sumudu transform can handle the complexities of FDPEs more effectively and offers a dynamic convergence to the appropriate solution faster and more precisely. On the contrary, RPSM might encounter difficulties or necessitate further modifications to manage fractional orders. Furthermore, several techniques are coupled with the Sumudu transformation approach, including the perturbation of transform technique^28^, Sumudu transform technique^29^, Variational sumudu strategy^30^. The residual power series method (RPSM) is one of the significant approach for investigating the computational results of various fractional problems in science and technology. Abu Arqub, a Jordanian mathematician, proposed the RPSM. Later, Wang and Chen^31^ utilized RPSM for the analytical results of temporal fractional Whitham–Broer–Kaup equations. Dubey et al.^32^ used RPSM for the approximate results of temporal fractional Black–Scholes problems. Tariq et al.^33^ applied the residual power series method to compute the results of (3+1)-dimensional NLSE with cubic nonlinearities. Korpinar and Inc^34^ used RPSM to compute the results of (1+1)-dimensional Biswas-Milovic problem of fractional variation which represents the long-space optical communications. Dubey and his colleagues^35^ utilized the efficiency of ST-RPSM for the solution of fractional Bloch equations appearing in an NMR flow. The RPSM can generate approximate analytic treatments for FDEs instead of utilizing linearization, variation, or discretization procedures, demonstrating credibility and elegance.

This work aims to illustrate the approximate results and their visualized observations for the time fractional NLSE by utilizing the Sumudu transform residual power series method (ST-RPSM). The acquired results are innovative, and the proposed technique is extremely effective in investigating the fractional model under consideration in this study. This scheme has several advantages. This method demonstrates the effectiveness and efficiency in achieving the expected outcomes. Variable discretization, large memory on a computer, or a long processing time are not needed to obtain the outcomes. Its nature is universal in terms of approximate analytical results, making it suitable for investigating a wide range of applications in mathematics and other scientific areas. The obtained solution of RPSM is extendable by using the commutations of iterations since they are analytical expressions. This paper has the following arrangements: Section "Preliminary concepts of fractional calculus and Sumudu transform" shows some concept of fractional calculus and Sumudu transform. The development of ST-RPSM step by step has been explained in Sect. "Formulation of ST-RPSM". We consider some numerical problems of time fractional NLSE in Sect. "Numerical Applications" and obtain their approximate results by using ST-RPSM. The summary of conclusion is presented in Sect. "Conclusion".

## Preliminary concepts of fractional calculus and Sumudu transform

This section covers the foundational discussion of some definitions of the Sumudu transform, fractional calculus, and RPSM. The subsequent definitions help in the construction of ST-RPSM.

### Definition 2.1

The Riemann-Liouville of order $\alpha > 0$ for expression of fractional integral operator is^36^

$$\begin{aligned} J^{\alpha }\mathfrak {S}(\wp )={\left\{ \begin{array}{ll} &{} \dfrac{1}{\Gamma (\alpha )}\int _{0}^{\wp }\mathfrak {S}(s)(\wp -s)^{\alpha -1}\ ds, \quad \alpha>0, \ \wp >0,\\ &{} \mathfrak {S}(\wp ), \alpha =0{.} \end{array}\right. } \end{aligned}$$

### Definition 2.2

The fractional derivative of $\mathfrak {S}(\aleph ,\wp )$ in Caputo form is^36^.

$$\begin{aligned} D^{\alpha }\mathfrak {S}(\aleph ,\wp )={\left\{ \begin{array}{ll} &{} \dfrac{1}{\Gamma (n-\alpha )}\int _{0}^{\wp }(\wp -q)^{n-\alpha -1}\dfrac{\partial ^{n}\mathfrak {S}(\aleph ,q)}{\partial q^{n}}\ dq{,} \qquad \qquad \quad m-1<\alpha < m,\\ &{} \partial ^{m}_{\wp }\mathfrak {S}(\aleph ,\wp )=\dfrac{\partial ^{m}\mathfrak {S}(\aleph ,\wp )}{\partial \wp ^{m}}, \qquad \qquad \alpha =m, \quad m\in \mathbb {N}. \end{array}\right. } \end{aligned}$$

### Definition 2.3

Let a series such as^37^

3 $$\begin{aligned} \sum _{m=0}^{\infty }\zeta _{m}(\wp -\wp _{0})^{m \alpha }=\zeta _{0}+\zeta _{1}(\wp -\wp _{0})^{\alpha }+\zeta _{2}(\wp -\wp _{0})^{2\alpha }+\cdots , \qquad \alpha>0, \quad \wp >\wp _{0}, \end{aligned}$$

which known as the power series in fractional form and $\wp =\wp _{0}$, where $\wp$ is a a parameter and $\zeta _{m}$ are set values in the solution of series function.

### Theorem 2.1

^37^ Consider $\zeta$ shows a power series in fractional form when $\wp =\wp _{0}$ in terms of

4 $$\begin{aligned} \zeta (\wp )=\sum _{m=0}^{\infty }\zeta _{m}(\wp -\wp _{0})^{m \alpha }, \end{aligned}$$

where $0<n-1<\alpha \le n,\ \aleph \in I,\ \wp _0 \le \wp <\wp _0+R$. If $D_\wp ^{m \alpha } \mathfrak {S}(\aleph , \wp )$ are continuous on $I \times (\wp _0, \wp _0+\mathbb {R}), m=$ $0,1,2, \cdots$, thus values for $\zeta _m(\aleph )$ are

5 $$\begin{aligned} \zeta _m(\aleph )=\frac{D_\wp ^{m \alpha } \mathfrak {S}(\aleph , \wp _0)}{\Gamma (m \alpha +1)}, \qquad m=0,1,2, \cdots , \end{aligned}$$

in which $$D_\wp ^{m \alpha }=D_\wp ^{\alpha }.D_\wp ^{\alpha }.\cdots D_\wp ^{\alpha } (n-times)$$.

### Proof

Let $\mathfrak {S}(\aleph , \wp )$ be a function of $\aleph$ and $\wp$, representing the power series of Eq. (3) in multiple fractional form. If we put $\wp =\wp _0$ in Eq. (4), then only its first component remains, and other components will vanish, so we obtain

6 $$\begin{aligned} \zeta _0(\aleph )=\mathfrak {S}\left( \aleph , \wp _0\right) . \end{aligned}$$

Applying the $D_\wp ^\alpha$ operator to Eq. (4), the subsequent expansion yields

7 $$\begin{aligned} D_\wp ^\alpha \mathfrak {S}(\aleph , \wp )=\Gamma (\alpha +1) \zeta _1(\aleph )+\frac{\Gamma (2 \alpha +1)}{\Gamma (\alpha +1)} \zeta _2(\aleph )\left( \wp -\wp _0\right) ^\alpha +\frac{\Gamma (3 \alpha +1)}{\Gamma (2 \alpha +1)} \zeta _3(\aleph )\left( \wp -\wp _0\right) ^{2 \alpha }+\ldots , \end{aligned}$$

On substituting $\wp =\wp _0$ to Eq. (7), we determine the value of $\zeta _1(\aleph )$ such as

8 $$\begin{aligned} \zeta _1(\aleph )=\frac{D_\wp ^\alpha \mathfrak {S}\left( \aleph , \wp _0\right) }{\Gamma (\alpha +1)} . \end{aligned}$$

Now, using an operator of $D_\wp ^\alpha$ one time on Eq. (7), the following expansion takes place:

9 $$\begin{aligned} D_0^{2 \alpha } \mathfrak {S}(\aleph , \wp )=\zeta _2 \Gamma (2 \alpha +1)+\zeta _3 \frac{\Gamma (3 \alpha +1)}{\Gamma (\alpha +1)}\left( \wp -\wp _0\right) ^\alpha +\zeta _4 \frac{\Gamma (4 \alpha +1)}{\Gamma (2 \alpha +1)}\left( \wp -\wp _0\right) ^{2 \alpha }+\ldots {.} \end{aligned}$$

On substituting $\wp =\wp _0$ to Eq. (9), we determine the value of $\zeta _2(\aleph )$ such as

10 $$\begin{aligned} \zeta _2(\aleph )=\frac{D_{\wp _0}^{2 \alpha } \mathfrak {S}\left( \aleph , \wp _0\right) }{\Gamma (2 \alpha +1)} . \end{aligned}$$

By repeating the operator $D_\wp ^\alpha$ *m* times and then using $\wp =\wp _0$, we can obtain the sequence of $\zeta _m(\aleph )$ such as:

11 $$\begin{aligned} \zeta _m(\aleph )=\frac{D_\wp ^{m \alpha } \mathfrak {S}\left( \aleph , \wp _0\right) }{\Gamma (m \alpha +1)}, \end{aligned}$$

which shows the agreement with Eq. (9). This proves the theorem.

*Note* By applying the series of $\zeta _m(\aleph )$ from Eq. (11) into Eq. (4), it is possible to obtain the power series of $\mathfrak {S}(\aleph , \wp )$ in multiple fractional form at the value of $\wp =\wp _0$ as follows,

12 $$\begin{aligned} \mathfrak {S}(\aleph , \wp )=\sum _{n=0}^{\infty } \frac{D_\wp ^{m \alpha } \mathfrak {S}\left( \aleph , \wp _0\right) }{\Gamma (m \alpha +1)}\left( \wp -\wp _0\right) ^{m \alpha }, \qquad n-1<\alpha \le n,\quad \wp _0 \le \wp <\wp _0+R, \end{aligned}$$

that represents the algorithm of Taylor’s formula in a generalized form. Now, if $\alpha =1$, Eq. (12) turns to Taylor’s series formula in a classical form such as

13 $$\begin{aligned} \mathfrak {S}(\aleph , \wp )=\sum _{n=0}^{\infty } \frac{\partial ^m \mathfrak {S}\left( \aleph , \wp _0\right) }{\partial \wp ^m} \frac{\left( \wp -\wp _0\right) ^m}{m !}, \qquad \wp _0 \le \wp <\wp _0+R{,} \end{aligned}$$

Thus, a new generalization form of Eq. (12) has been derived which helps to obtain the outcomes of time-fractional NLSE.

### Definition 2.4

The Sumudu transform on the set of functions *A* is expressed as^38^:

$$\begin{aligned} A=\mathfrak {S}(\wp ): \exists M, k_{1}, k_{2}>0, \mid \mathfrak {S}(\wp )\mid <M e^{\dfrac{\mid \wp \mid }{k_{j}}},\ \text{ if }\ \wp \in (-1)^{j}\times [0,\infty ), \end{aligned}$$

where *M* is constant for a finite parameter of a function in the set *A*, and $k_{1}, k_{2}$ may or may not be finite. Now, the integral relation of Sumudu transform is expressed as

14 $$\begin{aligned} S[\mathfrak {S}(\wp )]=R(\theta )=\int _{0}^{\infty } \mathfrak {S}(\theta \wp ) e^{-\wp } d \wp , \qquad \wp \ge 0, \quad k_{1}\le \theta \le k_{2}{.} \end{aligned}$$

The subsequent aspects of Sumudu transform are expressed as^39^:

1. $$S[\wp ^{n}]=n!\theta ^{n}$$,       $$n \in \mathbb {N}$$
2. $$S[\mathfrak {S}'(\wp )]=\dfrac{R(\theta )}{\theta }-\dfrac{\mathfrak {S}(0)}{\theta }$$,
3. $$S[\mathfrak {S}''(\wp )]=\dfrac{R(\theta )}{\theta ^{2}}-\dfrac{\mathfrak {S}(0)}{\theta ^{2}}-\dfrac{\mathfrak {S'}(0)}{\theta }$$,
4. $$S[\mathfrak {S}^{n}(\wp )]=\dfrac{R(\theta )}{\theta ^{n}}-\dfrac{\mathfrak {S}(0)}{\theta ^{n}}-\cdots -\dfrac{\mathfrak {S}^{n-1}(0)}{\theta }$$,
5. $$S[\wp ^{\alpha }]=\int _{0}^{\infty }e^{-\wp }\theta ^{\alpha }\ dt=\theta ^{\alpha }\Gamma (\alpha +1), \qquad n>-1$$.

### Definition 2.5

The fractional order of ST in Caputo sense is defined as^35^

15 $$\begin{aligned} S[D_{t}^{\alpha }\mathfrak {S}(\aleph , \wp )]=\theta ^{-\alpha } S[\mathfrak {S}(\aleph , \wp )]-\sum _{k=0}^{m-1} \theta ^{-\alpha +k} \mathfrak {S}^k(0, \wp ), \qquad m-1<\alpha <m{.} \end{aligned}$$

## Formulation of ST-RPSM

The present segment describes the formulation of ST-RPSM for the computational analysis of time-fractional NLSE. We observe that ST has the capability of changing fractional orders to a traditional space. This relation can easily be handled by RPSM to a system of algebraic equations which is close to the precise results of the fractional problems. We this formulation by a few steps where the complete procedure is described. Let $\mathfrak {S}(\aleph , \wp )$ and $\varrho (\aleph )$ be two complex functions with real and imaginary parts such that

16 $$\begin{aligned} \begin{aligned} \mathfrak {S}(\aleph , \wp )&=\vartheta (\aleph , \wp )+i \psi (\aleph , \wp ), \qquad \aleph \in \mathbb {R}, \quad \wp \ge 0{,}\\ \varrho (\aleph )&=\zeta (\aleph )+i \eta (\aleph ), \end{aligned} \end{aligned}$$

where $\vartheta (\aleph , \wp )$ and $\psi (\aleph , \wp )$ be two real valued functions over $\aleph \in \mathbb {R}$, $\zeta (\aleph )$ and $\eta (\aleph )$ are analytic functions on $\aleph \in \mathbb {R}$. By using Eq. (16), the system of Eq. (1) can be transformed to the following PDEs system such that

17 $$\begin{aligned} \begin{aligned} D_\wp ^\alpha \vartheta (\aleph , \wp )+\sigma \psi _{\aleph \aleph }(\aleph , \wp )+\gamma \left( \vartheta ^2(\aleph , \wp )+\psi ^2(\aleph , \wp )\right) \psi (\aleph , \wp )+\varsigma (\aleph ) \psi (\aleph , \wp )=0, \\ D_\wp ^\alpha \psi (\aleph , \wp )-\sigma \vartheta _{\aleph \aleph }(\aleph , \wp )-\gamma \left( \vartheta ^2(\aleph , \wp )+\psi ^2(\aleph , \wp )\right) \vartheta (\aleph , \wp )-\varsigma (\aleph ) \vartheta (\aleph , \wp )=0, \end{aligned} \end{aligned}$$

whereas the conditions becomes as

18 $$\begin{aligned} \begin{aligned} \vartheta (\aleph , 0)=\zeta (\aleph ),\\ \psi (\aleph , 0)=\eta (\aleph ). \end{aligned} \end{aligned}$$

Now, in general, the solution of Eq. (17) along condition (18) presents the solution of Eq. (1) with conditions (2). Hence, we develop the idea of ST-RPSM for Eq. (17) along condition (18). We will explain this concept with the following steps:

*Step 1* In our first stage, we execute the Sumudu transform to Eq. (17) and converting it to another space using condition (18), we get

19 $$\begin{aligned} \left\{ \begin{array}{l} \mathcal {P}(\aleph ,\theta )=\zeta (\aleph )-\theta ^{\alpha }S\Big [\sigma \psi _{\aleph \aleph }(\aleph , \wp )+\gamma \left( \vartheta ^2(\aleph , \wp )+\psi ^2(\aleph , \wp )\right) \psi (\aleph , \wp )+\varsigma (\aleph ) \psi (\aleph , \wp )\Big ], \\ ~\\ \mathcal {R}(\aleph ,\theta )=\eta (\aleph )+\theta ^{\alpha }S\Big [\sigma \vartheta _{\aleph \aleph }(\aleph , \wp )-\gamma \left( \vartheta ^2(\aleph , \wp )+\psi ^2(\aleph , \wp )\right) \vartheta (\aleph , \wp )-\varsigma (\aleph ) \vartheta (\aleph , \wp )\Big ], \end{array}\right. \end{aligned}$$

where $$\mathcal {P}(\aleph , \theta )=S\{\vartheta (\aleph , \wp )\}$$ and $$\mathcal {R}(\aleph , \theta )=S[\psi (\aleph , \wp )]$$.

*Step 2* In the second stage, Let the precise results of Eq. (19) for $\mathcal {P}(\aleph , \theta )$ and $\mathcal {R}(\aleph , \theta )$ have the subsequent expansions

20 $$\begin{aligned} \left\{ \begin{array}{l} \mathcal {P}(\aleph , \theta )=\sum _{n=0}^{\infty }\zeta _{n}(\aleph ) \theta ^{n\alpha }, \qquad 0<\alpha \le 1, \quad \theta>0,\\ ~\\ \mathcal {R}(\aleph , \theta )=\sum _{n=0}^{\infty }\eta _{n}(\aleph ) \theta ^{n\alpha }, \qquad 0<\alpha \le 1, \quad \theta >0. \end{array}\right. \end{aligned}$$

and the *k*-th truncated Sumudu series of Eq. (20) is expressed as

21 $$\begin{aligned} \left\{ \begin{array}{l} \mathcal {P}_{k}(\aleph , \theta )=\zeta (\aleph )+\sum _{n=1}^{k}{\zeta }_{n}(\aleph ) \theta ^{n\alpha },\qquad 0<\alpha \le 1, \quad \theta>0,\\ ~\\ \mathcal {R}_{k}(\aleph , \theta )=\eta (\aleph )+\sum _{n=1}^{k}\eta _{n}(\aleph ) \theta ^{n\alpha }, \qquad 0<\alpha \le 1, \quad \theta >0. \end{array}\right. \end{aligned}$$

One can determine the constants $\zeta _{n}$ and $\eta _{n}$ of the expansion series in Eq. (21) by calculating the *k*-th Sumudu residual functions.

*Step 3* At the third stage, we develop the residual formula, such that $Res^{1}$ and $Res^{2}$ show the iterative formula for Eq. (19) as follows

22 $$\begin{aligned} \left\{ \begin{array}{l} {S}{\text {Res}^{1}}(\aleph ,\theta )=\mathcal {P}(\aleph , \theta )-\zeta (\aleph )+\theta ^{\alpha }S\Big [\sigma \psi _{\aleph \aleph }(\aleph , \wp )+\gamma \left( \vartheta ^2(\aleph , \wp )+\psi ^2(\aleph , \wp )\right) \psi (\aleph , \wp )+\varsigma (\aleph ) \psi (\aleph , \wp )\Big ], \\ ~\\ {S}{\text {Res}^{1}}(\aleph ,\theta )=\mathcal {R}(\aleph , \theta )-\eta (\aleph )-\theta ^{\alpha }S\Big [\sigma \vartheta _{\aleph \aleph }(\aleph , \wp )-\gamma \left( \vartheta ^2(\aleph , \wp )+\psi ^2(\aleph , \wp )\right) \vartheta (\aleph , \wp )-\varsigma (\aleph ) \vartheta (\aleph , \wp )\Big ]. \end{array}\right. \end{aligned}$$

Hence, the *k*-th truncated Sumudu residual series for Eq. (22) becomes as

23 $$\begin{aligned} \left\{ \begin{array}{l} {S}({\text {Res}^{1}_{k}}(\aleph ,\theta ))=\mathcal {P}_{k}(\aleph , \theta )-\zeta (\aleph )-\theta ^{\alpha }S\Big [\sigma \psi _{k \aleph \aleph }(\aleph , \wp )+\gamma \left( \vartheta _{k}^2(\aleph , \wp )+\psi _{k}^2(\aleph , \wp )\right) \psi _{k}(\aleph , \wp )+\varsigma (\aleph ) \psi _{k}(\aleph , \wp )\Big ], \\ ~\\ {S}({\text {Res}^{1}_{k}}(\aleph ,\theta ))=\mathcal {R}_{k}(\aleph , \theta )-\eta (\aleph )+\theta ^{\alpha }S\Big [\sigma \vartheta _{k \aleph \aleph }(\aleph , \wp )-\gamma \left( \vartheta _{k}^2(\aleph , \wp )+\psi ^2(\aleph , \wp )\right) \vartheta _{k}(\aleph , \wp )-\varsigma (\aleph ) \vartheta _{k}(\aleph , \wp )\Big ]. \end{array}\right. \end{aligned}$$

There are some significant results from the RPSM such as

- $$\quad \displaystyle \lim _{k \rightarrow \infty } {S}({\text {Res}^{1}_{k}}(\aleph ,\theta ))={S}({\text {Res}^{1}}(\aleph ,\theta ))$$,       for $$\aleph \in I, \theta >\sigma \ge 0$$.
- $${S}({\text {Res}^{1}}(\aleph ,\theta ))=0$$,       for $$\aleph \in I, \theta >\sigma \ge 0$$.
- $$\quad \displaystyle \lim _{\theta \rightarrow \infty }\theta ^{k\alpha +1} {S}({\text {Res}^{1}_{k}}(\aleph ,\theta ))=0$$,       where $$\aleph \in I, \theta >\sigma \ge 0$$,    with $$k=1,2,3,\cdots$$

*Step 4* Put the *k*-th truncated Sumudu series of Eq. (21) to the $k-$th Sumudu residual function of Eq. (23).

**Step 5.** The coefficients of $\zeta _{k}(\aleph )$ and $\eta _{k}(\aleph )$ can be derived by using $\displaystyle \lim _{\theta \rightarrow \infty }\theta ^{k\alpha +1} {S}({\text {Res}^{1}_{k}}(\aleph ,\theta ))=0$ for $k=1,2,3, \cdots$. The derived coefficients are compiled in terms of an iterative series for $\mathcal {P}_{k}(\aleph , \theta )$ and $\mathcal {R}_{k}(\aleph , \theta )$ of the expansion (21).

*Step 6* By utilizing inverse ST to the obtained resultant series, we can achieve the approximate solution $\mathcal {P}_{k}(\aleph , \theta )$ and $\mathcal {R}_{k}(\aleph , \theta )$ of the main fractional problem (17).

## Numerical applications

In this segment, we show thevalidity, performance, and power of ST-RPSM by considering three applications of time fractional NLSE with different conditions. The Mathematica package has been utilized for performing complex conceptualization and computations involving mathematics.

### Problem 1

Consider one dimensional NLSE of time-fractional in the subsequent form^21,40^

24 $$\begin{aligned} i D_\wp ^\alpha \mathfrak {S}(\aleph , \wp )-\mathfrak {S}_{\aleph \aleph }(\aleph , \wp )=0, \qquad \aleph \in \mathbb {R}, \wp \ge 0,0<\alpha \le 1, \end{aligned}$$

in relates with following conditions

25 $$\begin{aligned} \mathfrak {S}(\aleph , 0)=e^{3 i \aleph }. \end{aligned}$$

The Eq. (24) can be simply transformed into the following fractional system:

26 $$\begin{aligned} \left\{ \begin{array}{l} D_\wp ^\alpha \vartheta (\aleph , \wp )-\psi _{\aleph \aleph }(\aleph , \wp )=0, \\ D_\wp ^\alpha \psi (\aleph , \wp )+\vartheta _{\aleph \aleph }(\aleph , \wp )=0, \end{array}\right. \end{aligned}$$

whereas the conditions becomes as

27 $$\begin{aligned} \vartheta (\aleph , 0)=\cos 3 \aleph , \qquad \psi (\aleph , 0)=\sin 3 \aleph . \end{aligned}$$

By applying ST to Eq. (26) along condition (27) and simplifying it, we get

28 $$\begin{aligned} \left\{ \begin{array}{l} \mathcal {P}(\aleph ,\theta )=\cos 3 \aleph +\theta ^{\alpha }\mathcal {R}_{\aleph \aleph }(\aleph ,\theta ), \\ ~\\ \mathcal {R}(\aleph ,\theta )=\sin 3 \aleph -\theta ^{\alpha }\mathcal {P}_{\aleph \aleph }(\aleph ,\theta ). \end{array}\right. \end{aligned}$$

Consider the precise result of Eq. (26) in the *k*-th expression such as

29 $$\begin{aligned} \left\{ \begin{array}{l} \mathcal {P}_{k}(\aleph ,\theta )=\cos 3 \aleph +\sum _{n=1}^{k}\zeta _{n}(\aleph ) \theta ^{n\alpha }, \\ ~\\ \mathcal {R}_{k}(\aleph ,\theta )=\sin 3 \aleph +\sum _{n=1}^{k}\eta _{n}(\aleph ) \theta ^{n\alpha }. \end{array}\right. \end{aligned}$$

Now, the *k*-th expression of Eq. (28) is also formed as

30 $$\begin{aligned} \left\{ \begin{array}{l} S({\text {Res}^{1}_{k}}(\aleph ,\theta ))=\mathcal {P}_{k}(\aleph ,\theta )-\cos 3 \aleph -\theta ^{\alpha }\mathcal {R}_{k \aleph \aleph }(\aleph ,\theta ), \\ ~\\ S({\text {Res}^{2}_{k}}(\aleph ,\theta ))=\mathcal {R}_{k}(\aleph ,\theta )-\sin 3 \aleph +\theta ^{\alpha }\mathcal {P}_{k \aleph \aleph }(\aleph ,\theta ). \end{array}\right. \end{aligned}$$

The 1st coefficient of Eq. (29) can be derived by utilizing the following truncated succession of first order

31 $$\begin{aligned} \left\{ \begin{array}{l} \mathcal {P}_{1}(\aleph ,\theta )=\cos 3 \aleph +\zeta _{1}(\aleph ) \theta ^{\alpha }, \\ ~\\ \mathcal {R}_{1}(\aleph ,\theta )=\sin 3 \aleph +\eta _{1}(\aleph ) \theta ^{\alpha }. \end{array}\right. \end{aligned}$$

By using using Eq. (31) into Eq. (30) at $k=1$, we obtain 1st ST-RPSM result

32 $$\begin{aligned} \left\{ \begin{array}{l} S({\text {Res}^{1}_{1}}(\aleph ,\theta ))=\zeta _{1}(\aleph ) \theta ^{\alpha }-\theta ^{\alpha }\Big (-9\sin 3\aleph +\eta ''_{1}\theta ^{\alpha }\Big ), \\ ~\\ S({\text {Res}^{2}_{1}}(\aleph ,\theta ))=\eta _{1}(\aleph ) \theta ^{\alpha }+\theta ^{\alpha }\Big (-9\cos 3\aleph +\zeta ''_{1}\theta ^{\alpha }\Big ) . \end{array}\right. \end{aligned}$$

Now, using the facts of RPSM such as limit of $\theta \rightarrow \infty$ for Eq. (32), we derive the results such as

33 $$\begin{aligned} \zeta _{1}(\aleph )=-9\sin 3\aleph , \qquad \eta _{1}(\aleph )=9\cos 3\aleph . \end{aligned}$$

Similarly, the 2nd coefficient of Eq. (29) can be derived by utilizing the following truncated succession of second order

34 $$\begin{aligned} \left\{ \begin{array}{l} \mathcal {P}_{2}(\aleph ,\theta )=\cos 3 \aleph -9 \sin 3\aleph \ \theta ^{\alpha } +\zeta _{2}\theta ^{2\alpha }, \\ ~\\ \mathcal {R}_{2}(\aleph ,\theta )=\sin 3 \aleph +9\cos 3\aleph \ \theta ^{\alpha } +\eta _{2}\theta ^{2\alpha }. \end{array}\right. \end{aligned}$$

By using using Eq. (34) into Eq. (30) at $k=2$, we obtain 2nd ST-RPSM result

35 $$\begin{aligned} \left\{ \begin{array}{l} S({\text {Res}^{1}_{2}}(\aleph ,\theta ))=\zeta _{2}(\aleph ) \theta ^{2\alpha }+81\theta ^{2\alpha }\cos 3\aleph -\eta ''_{2}\theta ^{4\alpha }\Big ), \\ ~\\ S({\text {Res}^{2}_{2}}(\aleph ,\theta ))=\eta _{2}(\aleph ) \theta ^{2\alpha }+81\theta ^{2\alpha }\sin 3\aleph +\zeta ''_{2}\theta ^{4\alpha }\Big ). \end{array}\right. \end{aligned}$$

Now, using the facts of RPSM such as limit of $\theta \rightarrow \infty$ for Eq. (35), we derive the results such as

36 $$\begin{aligned} \zeta _{2}(\aleph )=-81\cos 3\aleph , \qquad \eta _{2}(\aleph )=-81\sin 3\aleph . \end{aligned}$$

On similar way, we can derive the ST-RPSM results for $k=3,4$

37 $$\begin{aligned} \begin{aligned} \zeta _{3}(\aleph )=729\sin 3\aleph , \qquad \eta _{3}(\aleph )=-729\cos 3\aleph ,\\ \zeta _{4}(\aleph )=6561\cos 3\aleph , \qquad \eta _{4}(\aleph )=6561\sin 3\aleph . \end{aligned} \end{aligned}$$

Hence, the 4th ST-RPSM results of Eq. (29) can be expressed in the following series such as

38 $$\begin{aligned} \left\{ \begin{array}{l} \mathcal {P}_{4}(\aleph ,\theta )=\cos 3\aleph -9\sin 3\aleph \theta ^{\alpha }-81\cos 3xs^{2\alpha }+729\sin 3xs^{3\alpha }+6561\cos 3xs^{4\alpha }, \\ ~\\ \mathcal {R}_{4}(\aleph ,\theta )=\sin 3\aleph +9\cos 3\aleph \theta ^{\alpha }-81\sin 3xs^{2\alpha }-729\cos 3xs^{3\alpha }+6561\sin 3xs^{4\alpha }. \end{array}\right. \end{aligned}$$

By applying inverse ST on system of Eq. (38), we obtain

39 $$\begin{aligned} \left\{ \begin{array}{l} \vartheta _4(\aleph , \wp )=\cos (3 \aleph )-\dfrac{9 \sin (3 \aleph )}{\Gamma (1+\alpha )} \wp ^\alpha -\dfrac{(9)^2 \cos (3 \aleph )}{\Gamma (1+2 \alpha )} \wp ^{2 \alpha }+\dfrac{(9)^3 \sin (3 \aleph )}{\Gamma (1+3 \alpha )} \wp ^{3 \alpha }+\dfrac{(9)^4 \cos (3 \aleph )}{\Gamma (1+4 \alpha )} \wp ^{4 \alpha }, \\ ~\\ \psi _4(\aleph , \wp )=\sin (3 \aleph )+\dfrac{9 \cos (3 \aleph )}{\Gamma (1+\alpha )} \wp ^\alpha -\dfrac{(9)^2 \sin (3 \aleph )}{\Gamma (1+2 \alpha )} \wp ^{2 \alpha }-\dfrac{(9)^3 \cos (3 \aleph )}{\Gamma (1+2 \alpha )} \wp ^{3 \alpha }+\dfrac{(9)^4 \sin (3 \aleph )}{\Gamma (1+4 \alpha )} \wp ^{4 \alpha }. \end{array}\right. \end{aligned}$$

By using the similar process, this series can be continuous and thus ST-RPSM results for $\vartheta (\aleph , \wp )$ and $\psi (\aleph , \wp )$ can converge to the following form

40 $$\begin{aligned} \mathfrak {S}(\aleph , \wp )=e^{3 i \aleph }\chi (\wp ). \end{aligned}$$

whereas

$$\begin{aligned} \chi (\wp )=1+9i\frac{\wp ^{\alpha }}{\Gamma (1+\alpha )}+(9i)^{2}\frac{\wp ^{2\alpha }}{\Gamma (1+2\alpha )}+(9i)^{3}\frac{\wp ^{3\alpha }}{\Gamma (1+3\alpha )} +(9i)^{4}\frac{\wp ^{4\alpha }}{\Gamma (1+4\alpha )}+\cdots . \end{aligned}$$

Considering $\alpha =1$ in Eqs. (40) and (24) has the subsequent precise solution

41 $$\begin{aligned} \mathfrak {S}(\aleph , \wp )=e^{3 i (\aleph +3\wp )}, \end{aligned}$$

which has similar results with decomposition approach^22^, homotopy analysis method^41^, and variational approach^42^. Therefore, we can demonstrate that ST-RPSM is an easy, fundamental, and effective technique for solving fractional problems.

**Figure 1:**
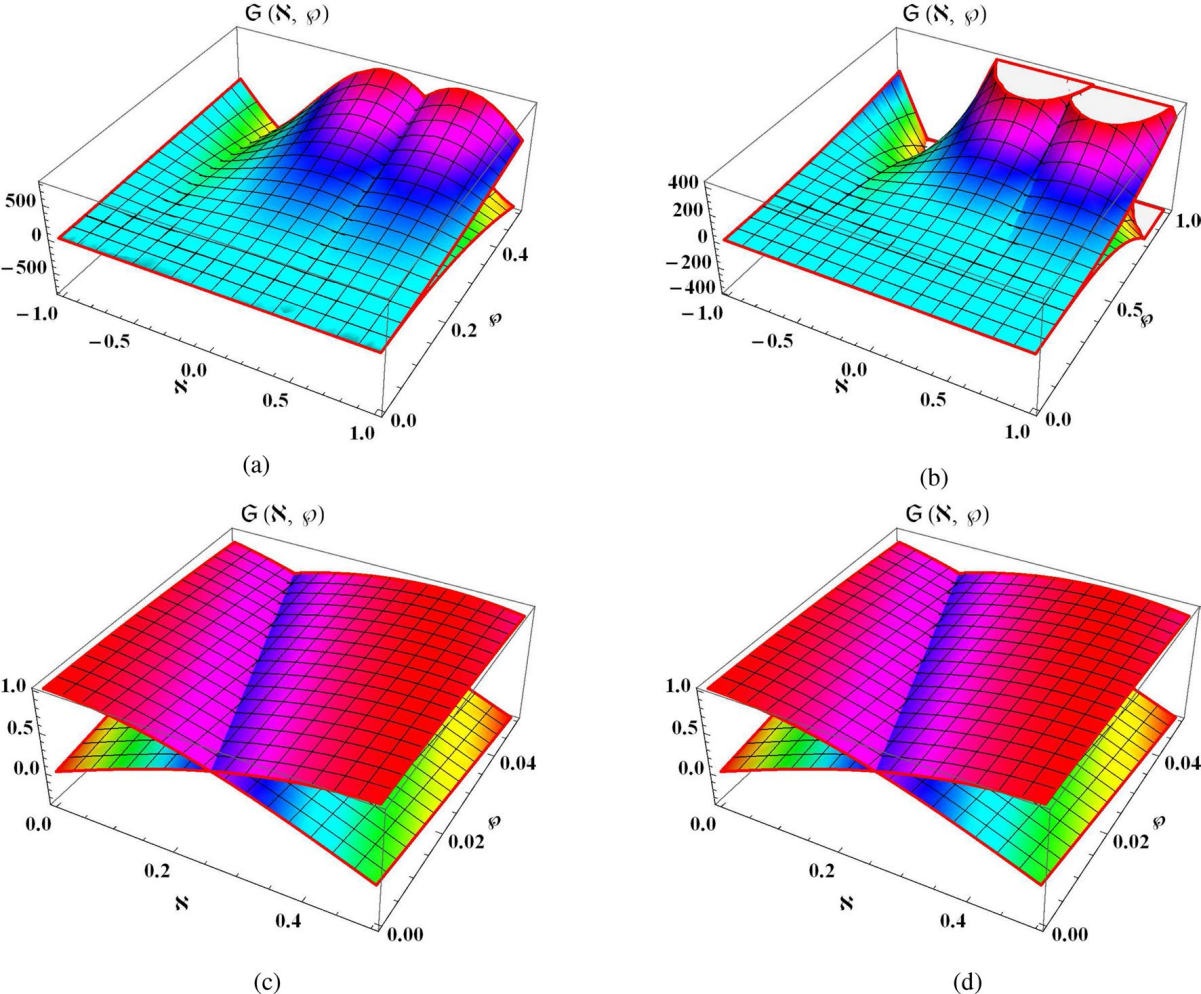
Visual framework of ST-RPSM results for $\vartheta (\aleph , \wp )$ and $\psi (\aleph , \wp )$.

Figure 1 demonstrates the graphical representation of time fractional NLSE in the shape of real and imaginary visuals and we divide it into its four graphical structures. Figure 1a shows the physical behavior of ST-RPSM solution in the form of 3D graphical structure by taking the fractional order at $\alpha =0.5$ with $-1\le \aleph \le 1, 0\le \wp \le 0.5$. Figure 1b shows the physical description of ST-RPSM solution in the form of 3D graphical structure by taking the fractional order at $\alpha =0.8$ with $-1\le \aleph \le 1, 0\le \wp \le 1$. Figure 1c illustrates the physical explanation of ST-RPSM outcomes in the form of 3D graphical structure by taking the fractional order at $\alpha =1$ with $0\le \aleph \le 0.5, 0\le \wp \le 0.05$. Figure 1d illustrates the physical assessment of precise results for NLSE in the form of 3D graphical structure at $0\le \aleph \le 0.5, 0\le \wp \le 0.05$. From these graphical structures, one can observe that time-fractional NSE has the full agreement of precise results with ST-RPSM outcomes at $\alpha =1$.

### Problem 2

Consider one dimensional NLSE of time-fractional in the subsequent form^21,40^

42 $$\begin{aligned} i D_\wp ^\alpha \mathfrak {S}(\aleph , \wp )+\mathfrak {S}_{\aleph \aleph }(\aleph , \wp )+2|\mathfrak {S}(\aleph , \wp )|^2 \mathfrak {S}(\aleph , \wp )=0, \qquad \aleph \in \mathbb {R},\ \wp \ge 0,\ 0<\alpha \le 1, \end{aligned}$$

in relates with following conditions

43 $$\begin{aligned} \mathfrak {S}(\aleph , 0)=e^{i \aleph }. \end{aligned}$$

The Eq. (42) can be simply transformed into the following fractional system:

44 $$\begin{aligned} \left\{ \begin{array}{l} D_\wp ^\alpha \vartheta (\aleph , \wp )+\psi _{\aleph \aleph }(\aleph , \wp )+2\left( \vartheta ^2(\aleph , \wp )+\psi ^2(\aleph , \wp )\right) \psi (\aleph , \wp )=0{,} \\ D_\wp ^\alpha \psi (\aleph , \wp )-\vartheta _{\aleph \aleph }(\aleph , \wp )-2\left( \vartheta ^2(\aleph , \wp )+\psi ^2(\aleph , \wp )\right) \vartheta (\aleph , \wp )=0, \end{array}\right. \end{aligned}$$

whereas the conditions becomes as

45 $$\begin{aligned} \vartheta (\aleph , 0)=\cos (\aleph ), \qquad \psi (\aleph , 0)=\sin (\aleph ). \end{aligned}$$

By applying ST to Eq. (44) along condition (45) and simplifying it, we get

46 $$\begin{aligned} \left\{ \begin{array}{l} \mathcal {P}(\aleph ,\theta )=\cos \aleph -\theta ^{\alpha }S\Big \{\mathcal {R}_{xx}(\aleph ,\theta )+2\Big (\mathcal {P}^{2}(\aleph ,\theta )+\mathcal {R}^{2}(\aleph ,\theta )\Big )\mathcal {R}(\aleph ,\theta )\Big \}, \\ ~\\ \mathcal {R}(\aleph ,\theta )=\sin \aleph +\theta ^{\alpha }S\Big \{\mathcal {P}_{xx}(\aleph ,\theta )+2\Big (\mathcal {P}^{2}(\aleph ,\theta )+\mathcal {R}^{2}(\aleph ,\theta )\Big )\mathcal {P}(\aleph ,\theta )\Big \}. \end{array}\right. \end{aligned}$$

Consider the precise result of Eq. (44) in the *k*-th expression such as

47 $$\begin{aligned} \left\{ \begin{array}{l} \mathcal {P}_{k}(\aleph ,\theta )=\cos \aleph +\sum _{n=1}^{k}\zeta _{n}(\aleph ) \theta ^{n\alpha }, \\ ~\\ \mathcal {R}_{k}(\aleph ,\theta )=\sin \aleph +\sum _{n=1}^{k}\eta _{n}(\aleph ) \theta ^{n\alpha }. \end{array}\right. \end{aligned}$$

Now, the *k*-th expression of Eq. (46) is also formed as

48 $$\begin{aligned} \left\{ \begin{array}{l} S({\text {Res}_{k}^{1}}(\aleph ,\theta ))=\mathcal {P}_{k}(\aleph ,\theta )-\cos \aleph +\theta ^{\alpha }S\Big \{\mathcal {R}_{k \aleph \aleph }(\aleph ,\theta )+2\Big (\mathcal {P}_{k}^{2}(\aleph ,\theta )+\Phi _{k}^{2}(\aleph ,\theta )\Big )\Phi _{k}(\aleph ,\theta )\Big \}, \\ ~\\ S({\text {Res}_{k}^{2}}(\aleph ,\theta ))=\mathcal {R}_{k}(\aleph ,\theta )-\sin \aleph -\theta ^{\alpha }S\Big \{\mathcal {P}_{k \aleph \aleph }(\aleph ,\theta )+2\Big (\mathcal {P}_{k}^{2}(\aleph ,\theta )+\mathcal {R}_{k}^{2}(\aleph ,\theta )\Big )\mathcal {P}_{k}(\aleph ,\theta )\Big \}. \end{array}\right. \end{aligned}$$

Using the ST-RPSM strategy, we can derive the results for $k=1,2,3,4$ such that

49 $$\begin{aligned} \begin{aligned} \zeta _1\aleph&=-\sin \aleph , \quad \qquad \quad \qquad \quad \qquad \quad \qquad \quad \qquad \quad \ \ \eta _1\aleph =\cos \aleph {,} \\ \zeta _2\aleph&=-\cos \aleph , \quad \qquad \quad \qquad \quad \qquad \quad \qquad \quad \qquad \quad \ \ \eta _2\aleph =-\sin \aleph {,}\\ \zeta _3\aleph&={ \left( 5-2 \frac{\Gamma (1+2 \alpha )}{\Gamma ^2(1+\alpha )}\right) \sin \aleph }, \quad \quad \quad \quad \qquad \quad \qquad \quad \qquad \ \ \eta _3\aleph ={-\left( 5-2 \frac{\Gamma (1+2 \alpha )}{\Gamma (1+\alpha )^2}\right) \cos \aleph }{,}\\ \zeta _4\aleph&={\left( 5-\frac{2 \Gamma (1+2 \alpha )}{\Gamma (1+\alpha )^2}+\frac{4 \Gamma (1+3 \alpha )}{\Gamma (1+\alpha ) \Gamma (1+2 \alpha )}-\frac{2 \Gamma (1+3 \alpha )}{\Gamma (1+\alpha )^3}\right) \cos \aleph }, \quad \eta _4\aleph ={\left( 5-\frac{2 \Gamma (1+2 \alpha )}{\Gamma (1+\alpha )^2}+\frac{4 \Gamma (1+3 \alpha )}{\Gamma (1+\alpha ) \Gamma (1+2 \alpha )}-\frac{2 \Gamma (1+3 \alpha )}{\Gamma (1+\alpha )^2}\right) \sin \aleph }. \end{aligned} \end{aligned}$$

Hence, the 4th ST-RPSM results of Eq. (47) can be expressed in the following series such as

50 $$\begin{aligned} \left\{ \begin{array}{l} \mathcal {P}_4(\aleph , \theta )=\cos \aleph -\theta ^{\alpha }\sin \aleph -\theta ^{2\alpha }\cos \aleph +\left( 5-2 \dfrac{\Gamma (1+2 \alpha )}{\Gamma ^2(1+\alpha )}\right) \theta ^{3\alpha }\sin \aleph \\ +\left( 5-\dfrac{2 \Gamma (1+2 \alpha )}{\Gamma (1+\alpha )^2}+\dfrac{4 \Gamma (1+3 \alpha )}{\Gamma (1+\alpha ) \Gamma (1+2 \alpha )}-\dfrac{2 \Gamma (1+3 \alpha )}{\Gamma (1+\alpha )^3}\right) \theta ^{4\alpha }\cos \aleph , \\ ~\\ \mathcal {R}_4(\aleph , \theta )=\sin \aleph +\theta ^{\alpha }\cos \aleph -\theta ^{2\alpha }\sin \aleph -\left( 5-2 \dfrac{\Gamma (1+2 \alpha )}{\Gamma ^2(1+\alpha )}\right) \theta ^{3\alpha }\cos \aleph \\ +\left( 5-\dfrac{2 \Gamma (1+2 \alpha )}{\Gamma (1+\alpha )^2}+\dfrac{4 \Gamma (1+3 \alpha )}{\Gamma (1+\alpha ) \Gamma (1+2 \alpha )}-\dfrac{2 \Gamma (1+3 \alpha )}{\Gamma (1+\alpha )^3}\right) \theta ^{4\alpha }\sin \aleph . \end{array}\right. \end{aligned}$$

By applying inverse ST on system of Eq. (50), we obtain

51 $$\begin{aligned} \left\{ \begin{array}{l} \vartheta _4(\aleph , \wp )=\cos \aleph -\sin \aleph \dfrac{\wp ^\alpha }{\Gamma (1+\alpha )}-\cos \aleph \dfrac{\wp ^{2 \alpha }}{\Gamma (1+2 \alpha )}+\left( 5-2 \dfrac{\Gamma (1+2 \alpha )}{\Gamma ^2(1+\alpha )}\right) \sin \aleph \dfrac{\wp ^{3 \alpha }}{\Gamma (1+3 \alpha )}\\ +\left( 5-\dfrac{2 \Gamma (1+2 \alpha )}{\Gamma (1+\alpha )^2}+\dfrac{4 \Gamma (1+3 \alpha )}{\Gamma (1+\alpha ) \Gamma (1+2 \alpha )}-\dfrac{2 \Gamma (1+3 \alpha )}{\Gamma (1+\alpha )^3}\right) \cos \aleph \dfrac{\wp ^{4 \alpha }}{\Gamma (1+4 \alpha )},\\ ~\\ \psi _4(\aleph , \wp )=\sin \aleph +\cos \aleph \dfrac{\wp ^\alpha }{\Gamma (1+\alpha )}-\sin \aleph \dfrac{\wp ^{2 \alpha }}{\Gamma (1+2 \alpha )}-\left( 5-2 \dfrac{\Gamma (1+2 \alpha )}{\Gamma (1+\alpha )^2}\right) \cos \aleph \dfrac{\wp ^{3 \alpha }}{\Gamma (1+2 \alpha )}\\ +\left( 5-\dfrac{2 \Gamma (1+2 \alpha )}{\Gamma (1+\alpha )^2}+\dfrac{4 \Gamma (1+3 \alpha )}{\Gamma (1+\alpha ) \Gamma (1+2 \alpha )}-\dfrac{2 \Gamma (1+3 \alpha )}{\Gamma (1+\alpha )^3}\right) \sin \aleph \dfrac{\wp ^{4 \alpha }}{\Gamma (1+4 \alpha )}, \end{array}\right. \end{aligned}$$

By using the similar process, this series can be continuous and thus ST-RPSM results for $\vartheta (\aleph , \wp )$ and $\psi (\aleph , \wp )$ can converge to the following form

52 $$\begin{aligned} \mathfrak {S}(\aleph , \wp )=e^{i \aleph }\chi (\wp ). \end{aligned}$$

whereas

$$\begin{aligned} \chi (\wp )=1-3i\frac{\wp ^{\alpha }}{\Gamma (1+\alpha )}+(3i)^{2}\frac{\wp ^{2\alpha }}{\Gamma (1+2\alpha )}-i^{3}\Big (63-\frac{18\Gamma (1+2\alpha )}{\Gamma ^{2}(1+\alpha )}\Big ) \frac{\wp ^{3\alpha }}{\Gamma (1+3\alpha )}+\cdots . \end{aligned}$$

Considering $\alpha =1$ in Eq. (52), the system of Eqs. (24) along (25) has the subsequent precise result

53 $$\begin{aligned} \mathfrak {S}(\aleph , \wp )=e^{i (\aleph +\wp )}, \end{aligned}$$

which has similar results with decomposition approach^22^, homotopy analysis method^41^, and variational approach^42^. Therefore, we can demonstrate that ST-RPSM is an easy, fundamental, and effective technique for solving fractional problems.

**Figure 2:**
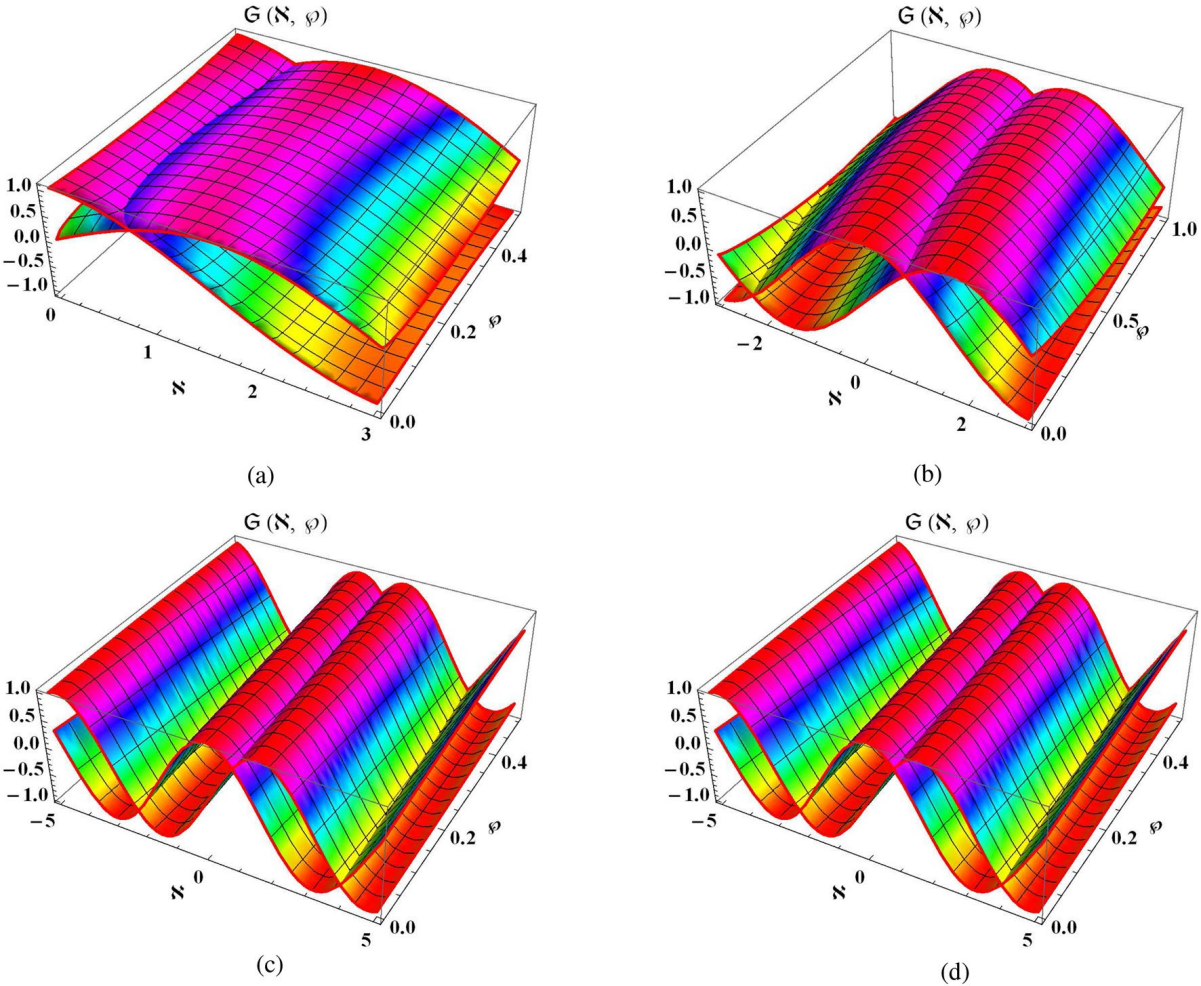
Visual framework of ST-RPSM results for $\vartheta (\aleph , \wp )$ and $\psi (\aleph , \wp )$.

Figure 2 demonstrates the graphical representation of time fractional NLSE in the shape of real and imaginary visuals and we divide it into its four graphical structures. Figure 2a shows the physical behavior of ST-RPSM solution in the form of 3D graphical structure by taking the fractional order at $\alpha =0.5$ with $0\le \aleph \le 3, 0\le \wp \le 0.5$. Figure 2b shows the physical description of ST-RPSM solution in the form of 3D graphical structure by taking the fractional order at $\alpha =0.8$ with $-3\le \aleph \le 3, 0\le \wp \le 1$. Figure 2c illustrates the physical explanation of ST-RPSM outcomes in the form of 3D graphical structure by taking the fractional order at $\alpha =1$ with $-5\le \aleph \le 5, 0\le \wp \le 0.5$. Figure 2d illustrates the physical assessment of precise results for NLSE in the form of 3D graphical structure at $\alpha =1$ with $-5\le \aleph \le 5, 0\le \wp \le 0.5$. From these graphical structures, one can observe that time-fractional NSE has the full agreement of precise results with ST-RPSM outcomes at $\alpha =1$.

### Problem 3

Consider one dimensional NLSE of time-fractional in the subsequent form^21,40^

54 $$\begin{aligned} i D_\wp ^\alpha \mathfrak {S}(\aleph , \wp )+\mathfrak {S}_{\aleph \aleph }(\aleph , \wp )+2|\mathfrak {S}(\aleph , \wp )|^4 \mathfrak {S}(\aleph , \wp )=0, \qquad \aleph \in \mathbb {R},\quad \wp \ge 0,\quad 0<\alpha \le 1, \end{aligned}$$

in relates with following conditions

55 $$\begin{aligned} \mathfrak {S}(\aleph , 0)=(6 {{\,\textrm{sech}\,}}^{2}(4\aleph ))^{\frac{1}{4}}. \end{aligned}$$

The Eq. (54) can be simply transformed into the following fractional system:

56 $$\begin{aligned} \left\{ \begin{array}{l} D_\wp ^\alpha \vartheta (\aleph , \wp )+\psi _{\aleph \aleph }(\aleph , \wp )+2\left( \vartheta ^4(\aleph , \wp )+2 \vartheta ^2(\aleph , \wp ) \psi ^2(\aleph , \wp )+\psi ^4(\aleph , \wp )\right) \psi (\aleph , \wp )=0, \\ D_\wp ^\alpha \psi (\aleph , \wp )-\vartheta _{\aleph \aleph }(\aleph , \wp )-2\left( \vartheta ^4(\aleph , \wp )+2 \vartheta ^2(\aleph , \wp ) \psi ^2(\aleph , \wp )+\psi ^4(\aleph , \wp )\right) \vartheta (\aleph , \wp )=0, \end{array}\right. \end{aligned}$$

whereas the conditions becomes as

57 $$\begin{aligned} \vartheta (\aleph , 0)=\left( 6 {\text {sech}}^2(4 \aleph )\right) ^{\frac{1}{4}}, \qquad \psi (\aleph , 0)=0{.} \end{aligned}$$

By applying ST to Eq. (56) along condition (57) and simplifying it, we get

58 $$\begin{aligned} \left\{ \begin{array}{l} \mathcal {P}(\aleph ,\theta )=(6 sech^{2}(4\aleph ))^{\frac{1}{4}}-\theta ^{\alpha }S\Big \{\mathcal {R}_{xx}(\aleph ,\theta )+2\Big (\mathcal {P}^{4}(\aleph ,\theta )+2\mathcal {P}^{2}(\aleph ,\theta )\mathcal {R}^{2}(\aleph ,\theta )+\mathcal {R}^{4}(\aleph ,\theta )\Big )\mathcal {R}(\aleph ,\theta )\Big \}, \\ ~\\ \mathcal {R}(\aleph ,\theta )=\theta ^{\alpha }S\Big \{\mathcal {P}_{xx}(\aleph ,\theta )+2\Big (\mathcal {P}^{4}(\aleph ,\theta )+2\mathcal {P}^{2}(\aleph ,\theta )\mathcal {R}^{2}(\aleph ,\theta )+\mathcal {R}^{4}(\aleph ,\theta )\Big )\mathcal {P}(\aleph ,\theta )\Big \}. \end{array}\right. \end{aligned}$$

Let the precise result of Eq. (56) in *k*-th function is expressed as

59 $$\begin{aligned} \left\{ \begin{array}{l} \mathcal {P}_{k}(\aleph ,\theta )=\left( 6 {\text {sech}}^2(4 \aleph )\right) ^{\frac{1}{4}}+\sum _{n=1}^{k}\zeta _{n}(\aleph ) \theta ^{n\alpha }, \\ ~\\ \mathcal {R}_{k}(\aleph ,\theta )=\sum _{n=1}^{k}\eta _{n}(\aleph ) \theta ^{n\alpha }. \end{array}\right. \end{aligned}$$

Now, the *k*-th expression of Eq. (58) is also formed as

60 $$\begin{aligned} \left\{ \begin{array}{l} S({\text {Res}_{k}^{1}}(\aleph ,\theta ))=\mathcal {P}_{k}(\aleph ,\theta )-\left( 6 {\text {sech}}^2(4 \aleph )\right) ^{\frac{1}{4}}+\theta ^{\alpha }S\Big \{\mathcal {R}_{k \aleph \aleph }(\aleph ,\theta )+2\Big (\mathcal {P}_{k}^{4}(\aleph ,\theta )+2\mathcal {P}_{k}^{2}(\aleph ,\theta )\mathcal {R}_{k}^{2}(\aleph ,\theta )+\mathcal {R}_{k}^{4}(\aleph ,\theta )\Big )\mathcal {R}_{k}(\aleph ,\theta )\Big \}, \\ ~\\ S({\text {Res}_{k}^{2}}(\aleph ,\theta ))=\mathcal {R}_{k}(\aleph ,\theta )-\theta ^{\alpha }S\Big \{\mathcal {P}_{k \aleph \aleph }(\aleph ,\theta )+2\Big (\mathcal {P}_{k}^{4}(\aleph ,\theta )+2\mathcal {P}_{k}^{2}(\aleph ,\theta )\mathcal {R}_{k}^{2}(\aleph ,\theta )+\mathcal {R}_{k}^{4}(\aleph ,\theta )\Big )\mathcal {P}_{k}(\aleph ,\theta )\Big \}. \end{array}\right. \end{aligned}$$

Using the ST-RPSM strategy, we can derive the results for $k=1,2,3,4$ such that

61 $$\begin{aligned} \begin{aligned} \zeta _1(\aleph )&=0, \quad \qquad \quad \qquad \quad \qquad \quad \qquad \quad \qquad \quad \qquad \quad \qquad \quad \quad \eta _1(\aleph )=4\left( 6 {\text {sech}}^2(4 \aleph )\right) ^{\frac{1}{4}}{,} \\ \zeta _2(\aleph )&=-4^{2}\left( 6 {\text {sech}}^2(4 \aleph )\right) ^{\frac{1}{4}}, \quad \qquad \quad \qquad \quad \qquad \quad \qquad \quad \qquad \quad \ \ \eta _2(\aleph )=0{,}\\ \zeta _3(\aleph )&=0, \quad \quad \quad \quad \qquad \quad \qquad \quad \qquad \qquad \eta _3(\aleph )=-4^{3}\left( 6 {\text {sech}}^2(4 \aleph )\right) ^{\frac{1}{4}}\Bigg (\Big (\frac{25}{2}+\frac{1}{2}\cosh 8 \aleph -\frac{6\Gamma (1+2\alpha )}{\Gamma (1+\alpha )^{2}}\Big ){{\,\textrm{sech}\,}}^{2} 4 \aleph \Bigg ){,}\\ \zeta _4(\aleph )&=4^{4}\left( 6 {\text {sech}}^2(4 \aleph )\right) ^{\frac{1}{4}}\Bigg (\frac{601}{2}+6\frac{\Gamma (1+3\alpha )}{\Gamma (1+\alpha )^{3}}\Big (\frac{2\Gamma (1+\alpha )^{2}}{\Gamma (1+2\alpha )}-1\Big ) \\ {}&+\frac{1}{2}\cosh 8 \aleph -384{{\,\textrm{sech}\,}}^{2} 4 \aleph +\frac{6\Gamma (1+2\alpha )}{\Gamma (1+\alpha )^{2}}\Big (32{{\,\textrm{sech}\,}}^{2} 4 \aleph -25\Big )\Bigg ){{\,\textrm{sech}\,}}^{2} 4 \aleph , \quad \eta _4(\aleph )=0. \end{aligned} \end{aligned}$$

Hence, the 4th ST-RPSM results of Eq. (59) can be expressed in the following series such as

62 $$\begin{aligned} \left\{ \begin{array}{l} \mathcal {P}_4(\aleph , \theta )=(6 {{\,\textrm{sech}\,}}^{2}(4\aleph ))^{\frac{1}{4}}+(4i)^{2}\Big (6 {{\,\textrm{sech}\,}}^{2}(4\aleph )\Big )^{\frac{1}{4}}\theta ^{2\alpha }+4^{4}\left( 6 {\text {sech}}^2(4 \aleph )\right) ^{\frac{1}{4}}\theta ^{4\alpha }\Bigg (\frac{601}{2}+6\frac{\Gamma (1+3\alpha )}{\Gamma (1+\alpha )^{3}}\Big (\frac{2\Gamma (1+\alpha )^{2}}{\Gamma (1+2\alpha )}-1\Big ) \\ +\frac{1}{2}\cosh 8 \aleph -384{{\,\textrm{sech}\,}}^{2} 4 \aleph +\frac{6\Gamma (1+2\alpha )}{\Gamma (1+\alpha )^{2}}\Big (32{{\,\textrm{sech}\,}}^{2} 4 \aleph -25\Big )\Bigg ){{\,\textrm{sech}\,}}^{2} 4 \aleph , \\ ~\\ \mathcal {R}_4(\aleph , \theta )=4\Big (6 {{\,\textrm{sech}\,}}^{2}(4\aleph )\Big )^{\frac{1}{4}}\theta ^{\alpha }-4^{3}\left( 6 {{\,\textrm{sech}\,}}^2(4 \aleph )\right) ^{\frac{1}{4}}\theta ^{3\alpha }\Bigg (\Big (\frac{25}{2}+\frac{1}{2}\cosh 8 \aleph -\frac{6\Gamma (1+2\alpha )}{\Gamma (1+\alpha )^{2}}\Big ){{\,\textrm{sech}\,}}^{2} 4 \aleph \Bigg ). \end{array}\right. \end{aligned}$$

By applying inverse ST on system of Eq. (62), we obtain

63 $$\begin{aligned} \left\{ \begin{array}{l} \vartheta _4(\aleph , \wp )=(6 {{\,\textrm{sech}\,}}^{2}(4\aleph ))^{\frac{1}{4}}+(4i)^{2}\Big (6 {{\,\textrm{sech}\,}}^{2}(4\aleph )\Big )^{\frac{1}{4}}\dfrac{\wp ^{2\alpha }}{\Gamma (1+2\alpha )}+4^{4}\left( 6 {{\,\textrm{sech}\,}}^2(4\aleph )\right) ^{\frac{1}{4}}\Bigg (\frac{601}{2}+6\frac{\Gamma (1+3\alpha )}{\Gamma (1+\alpha )^{3}}\Big (\frac{2\Gamma (1+\alpha )^{2}}{\Gamma (1+2\alpha )}-1\Big ) \\ +\frac{1}{2}\cosh 8 \aleph -384{{\,\textrm{sech}\,}}^{2} 4 \aleph +\frac{6\Gamma (1+2\alpha )}{\Gamma (1+\alpha )^{2}}\Big (32{{\,\textrm{sech}\,}}^{2} 4 \aleph -25\Big )\Bigg ){{\,\textrm{sech}\,}}^{2} 4 \aleph \dfrac{\wp ^{4\alpha }}{\Gamma (1+4\alpha )}, \\ ~\\ \psi _4(\aleph , \wp )=4\Big (6 {{\,\textrm{sech}\,}}^{2}(4\aleph )\Big )^{\frac{1}{4}}\dfrac{\wp ^{\alpha }}{\Gamma (1+\alpha )}-4^{3}\left( 6 {{\,\textrm{sech}\,}}^2(4 \aleph )\right) ^{\frac{1}{4}}\Bigg (\Big (\frac{25}{2}+\frac{1}{2}\cosh 8 \aleph -\frac{6\Gamma (1+2\alpha )}{\Gamma (1+\alpha )^{2}}\Big ){{\,\textrm{sech}\,}}^{2} 4 \aleph \Bigg )\dfrac{\wp ^{3\alpha }}{\Gamma (1+3\alpha )}. \end{array}\right. \end{aligned}$$

By using the similar process, this series can be continuous and thus ST-RPSM results for $\vartheta (\aleph , \wp )$ and $\psi (\aleph , \wp )$ can converge to the following form

64 $$\begin{aligned} \begin{aligned} \mathfrak {S}(\aleph , \wp )&=\Big (6 {{\,\textrm{sech}\,}}^{2}(4\aleph )\Big )^{\frac{1}{4}}\Bigg (1+4i \frac{\wp ^{\alpha }}{\Gamma (1+\alpha )}+(4i)^{2} \frac{\wp ^{2\alpha }}{\Gamma (1+2\alpha )}\\ {}&+(4i)^{3}\Big (\frac{25}{2}+\frac{1}{2}\cosh 8 \aleph -\frac{6\Gamma (1+2\alpha )}{\Gamma (1+\alpha )^{2}}\Big ){{\,\textrm{csch}\,}}^{2} 4 \aleph \frac{\wp ^{3\alpha }}{\Gamma (1+3\alpha )}+\cdots \Bigg ). \end{aligned} \end{aligned}$$

Considering $\alpha =1$ in Eq. (64), Eq. (54) has the subsequent precise solution

65 $$\begin{aligned} \mathfrak {S}(\aleph , \wp )=\Big (6 {{\,\textrm{sech}\,}}^{2}(4\aleph )\Big )^{\frac{1}{4}}e^{4 i \aleph }, \end{aligned}$$

which has similar results with decomposition approach^22^, homotopy analysis method^41^, and variational approach^42^. Therefore, we can demonstrate that ST-RPSM is an easy, fundamental, and effective technique for solving fractional problems.

**Figure 3:**
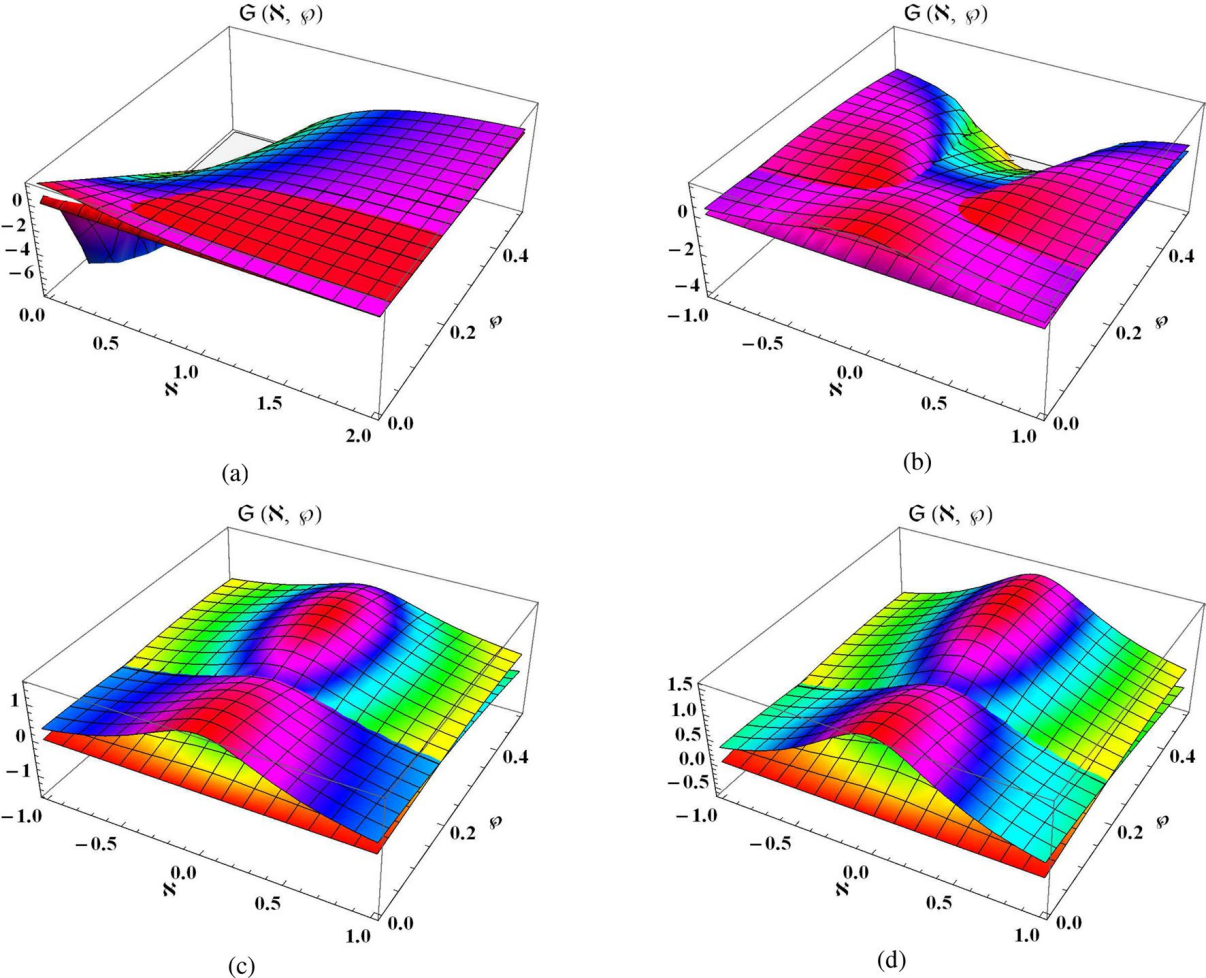
Visual framework of ST-RPSM results for $\vartheta (\aleph , \wp )$ and $\psi (\aleph , \wp )$.

Figure 3 demonstrates the graphical representation of time fractional NLSE in the shape of real and imaginary visuals and we divide it into its four graphical structures. Figure 3a shows the physical behavior of ST-RPSM solution in the form of 3D graphical structure by taking the fractional order at $\alpha =0.5$ with $0\le \aleph \le 2, 0\le \wp \le 0.5$. Figure 3b shows the physical description of ST-RPSM solution in the form of 3D graphical structure by taking the fractional order at $\alpha =0.8$ with $-1\le \aleph \le 1, 0\le \wp \le 0.5$. Figure 3c illustrates the physical explanation of ST-RPSM outcomes in the form of 3D graphical structure by taking the fractional order at $\alpha =1$ with $-1\le \aleph \le 1, 0\le \wp \le 0.5$. Figure 3d illustrates the physical assessment of precise results for NLSE in the form of 3D graphical structure at $\alpha =1$ with $-1\le \aleph \le 1, 0\le \wp \le 0.5$. From these graphical structures, one can observe that time-fractional NLSE has the full agreement of precise results with ST-RPSM outcomes when $\alpha =1$.

## Conclusion

In this work, we successfully obtained the approximate solutions of numerical results of time-fractional NLSE by utilizing the composition of Sumudu transform and residual power series scheme. The suggested approach yields the series results in a convergence form with minimal computational iterations. The Sundum transform can transfer the fractional order into a recurrence relation and the residual power series scheme can easily derive the series results from an algebraic system of fractional equations. The residual power series scheme has an excellent ability to handle nonlinear problems in time fractional models. We demonstrated the effectiveness and dependability of the suggested approach with three nonlinear models under Caputo fractional derivatives. A graphic illustration is used for analyzing the calculated results. The solutions provided to the time-fractional NLSE indicate the wave function of a quantum framework. Numerical computations are produced to show the graphical depictions of these wave functions, which offer valuable insights into the spatial dispersion of the quantum particle as it expands over time. These visualizations aid in comprehending the concept of the duality of wave particles and the uncertain characteristics of quantum physics. The derived results show that the obtained series results for different levels of fractional order confirm the reliability, comparability, and simplicity of the time-fractional NLSE in numerous branches of science and technology. In future work, we extend this scheme to investigate the ordinary and partial differential equations with fractional derivatives in nonlinear models of science and technology having exponential and Mittag-Leffler kernels.

## Data Availability

All data generated or analysed during this study are included in this published article.
